# Supplementary material for: Feasibility study of a randomised controlled trial to investigate the treatment of sarcoidosis-associated fatigue with methylphenidate (FaST-MP): a study protocol
Source: BMJ Open. 2017 Dec 4;7(12):e018532. doi: 10.1136/bmjopen-2017-018532 (PMC5719286; doi:10.1136/bmjopen-2017-018532)
Supplement: Author's manuscript [file bmjopen-2017-018532.draft_revisions.pdf]

BMJ Open is committed to open peer review. As part of this commitment we make the peer review history of every article we publish publicly available.

When an article is published we post the peer reviewers' comments and the authors' responses online. We also post the versions of the paper that were used during peer review. These are the versions that the peer review comments apply to.

The versions of the paper that follow are the versions that were submitted during the peer review process. They are not the versions of record or the final published versions. They should not be cited or distributed as the published version of this manuscript.

BMJ Open is an open access journal and the full, final, typeset and author-corrected version of record of the manuscript is available on our site with no access controls, subscription charges or pay-per-view fees (http://bmjopen.bmj.com).

If you have any questions on BMJ Open's open peer review process please email editorial.bmjopen@bmj.com

BMJ Open

Feasibility study of a randomised controlled trial to investigate the treatment of sarcoidosis-associated fatigue with methylphenidate (FaST-MP): a study protocol

Journal:	*BMJ Open*
Manuscript ID	bmjopen-2017-018532
Article Type:	Protocol
Date Submitted by the Author:	05-Jul-2017
Complete List of Authors:	Atkins, Christopher; University of East Anglia Norwich Medical School, Department of Medicine; University of East Anglia Norwich Medical School, Department of Medicine<br>Fordham, Richard; University of East Anglia Norwich Medical School, Department of Medicine<br>Clark, Allan; University of East Anglia Norwich Medical School, Department of Medicine<br>Stockl, Andrea; University of East Anglia Norwich Medical School, Department of Medicine<br>Wilson, Andrew; University of East Anglia Norwich Medical School, Department of Medicine
**Primary Subject Heading**:	Respiratory medicine
Secondary Subject Heading:	Research methods, Pharmacology and therapeutics
Keywords:	Interstitial lung disease < THORACIC MEDICINE, Sarcoidosis, Fatigue

SCHOLARONE™
Manuscripts

For peer review only

Feasibility study of a randomised controlled trial to investigate the treatment of sarcoidosis-associated fatigue with methylphenidate (FaST-MP): a study protocol

[1,2]C Atkins[a], [1]R Fordham, [1]A Clarke, [1]A Stockl, [1,2]A M Wilson

[1]Norwich Medical School, University of East Anglia, Norwich, Norfolk, UK, NR4 7TJ

[2]Norfolk and Norwich University Hospital, Norwich, Norfolk, UK, NR4 7UY

[a]Corresponding author: Dr Chris Atkins, Norwich Medical School, University of East Anglia, Norwich, Norfolk, UK, NR4 7TJ

E-mail contact: c.atkins@uea.ac.uk

For peer review only

Abstract

Introduction

Fatigue is a frequent and troublesome manifestation of chronic sarcoidosis. This symptom can be debilitating and difficult to treat with poor response to treatment. Symptomatic management with neurostimulants such as methylphenidate is one possible treatment option in but has little evidence. The use of such treatment strategies is not without precedent and has been trialled in cancer-related fatigue. Use in sarcoidosis requires further evaluation before it can be recommended for clinical practice.

Methods and Analysis

The Fatigue and Sarcoidosis – Treatment with Methylphenidate (FaST-MP) study is a randomised controlled trial of methylphenidate for the treatment of sarcoidosis-associated fatigue. Patients are eligible if they have a diagnosis of sarcoidosis, significant fatigue (measured using the Fatigue Assessment Scale) and have stable disease. Up to 30 participants will be randomly assigned to either methylphenidate (20mg twice daily) or identical placebo in a 3:2 ratio for 24 weeks. The primary objective is to collect data determining the feasibility of a future study powered to determine the clinical efficacy of methylphenidate for sarcoidosis-associated fatigue. The trial is presently open and will continue until July 2018.

Ethics and Dissemination

Ethical approval for the study was granted by the Cambridge Central Research Ethics Committee on 21 June 2016 (reference 16/EE/0087) and was approved and sponsored by the Norfolk and Norwich University Hospital (reference 190280). Clinical Trial Authorisation (EudraCT number 2016-000342-60) from the MHRA was granted on 19 April 2016. Results will be presented at relevant conferences and submitted to appropriate journals following trial closure and analysis.

Trial registration: NCT02643732

For peer review only

Article Summary

Strengths and limitations of this study:

- Twenty-four week duration of receiving methylphenidate or placebo
- Variety of outcome measures included to inform future study design
- Exercise measurements during study done both in clinic (modified shuttle walk test) and using objective measures of exercise during free-living (wrist-worn activity monitors)
- No stratification for age or sex due to small number of participants

For peer review only

Introduction

Sarcoidosis is a granulomatous, multi-system disease with an incidence of 5 per 100,000 person-years, typically affecting patients of working age(1). Fatigue has been described as a "core symptom" of sarcoidosis and is present in up to 80% of patients (2, 3). The presence of fatigue has shown to adversely affect quality of life(4). Sarcoidosis-associated fatigue has multiple possible aetiologies. These include subclinical disease activity(5), increased circulating tumour necrosis factor (TNF)-alpha levels(6), disturbed circadian rhythm(7) and sleep(8), small-fibre neuropathy(9), depression(10), sleep-disordered breathing problems(11), treatment-related side-effects (12), or a combination of any of these factors.

Role of neurostimulants in treating fatigue

Current evidence for treatments of sarcoidosis-associated fatigue are weak, with further work required (13). The use of methylphenidate for fatigue is not without precedent, having been trialled in cancer-related fatigue and recommended as effective for symptomatic relief after meta-analysis by the Cochrane collaboration(14). There is also evidence for effect in fatigue associated with Parkinson's disease(15) and HIV(16). In all cases, the treatment has only been trialled for a short period of time (up to 12 weeks). Neurostimulants have been trialled for symptomatic relief of fatigue associated with sarcoidosis. Both methylphenidate(17) and modafinil(18) have been trialled in patients with significant fatigue, though in only a very small sample (10 patients trialled methylphenidate and 15 trialled modafinil) over only eight weeks, a period of time that does not reflect the typical period of time that these agents may be used for. Therefore questions remain about ongoing treatment effect and whether patients would continue to take the medication over a longer period.

Rationale for this pilot trial

High quality evidence is required to establish the clinical efficacy and long-term tolerability of methylphenidate for the treatment of sarcoidosis-associated fatigue. Change in fatigue scores, measured by a validated questionnaire measure of fatigue across a longer treatment period of six months would be able to address the issue of efficacy, but the evidence to support a large-scale randomised controlled trial (RCT) is presently insufficient.

Furthermore, although recommendations from the World Association of Sarcoidosis and Other Granulomatous diseases (WASOG) recommend measuring fatigue in trials involving sarcoidosis patients using a validated questionnaire(19), we plan to also investigate the effect on activity levels with treatment using wrist-worn accelerometers alongside typical questionnaire measures. Outputs from these activity monitors appear to correlate with fatigue scores, both in terms of reduced activity and increased time in sedentary behaviours (unpublished data), and may give further insight into how improvements in fatigue affect day-to-day living in patients. To plan a sufficiently large RCT we propose a pilot placebo-controlled RCT to inform the design of a future, definitive trial investigating management of fatigue.

Objectives

The primary hypothesis is to determine the feasibility and design of a future, large-scale RCT investigating the use of methylphenidate to treat sarcoidosis-associated fatigue and provide sustained benefit over a six-month period. In addition, it will pilot the use of wrist-worn, tri-axial accelerometer-based activity monitors to monitor change in activity and sedentary behaviours during treatment alongside validated questionnaire-based fatigue scores.

Methods

For peer review only

Participants

This study is a randomised, double-blind, placebo-controlled single-centre parallel-group trial with randomisation of up to 30 patients in a ratio of 3:2 in favour of methylphenidate. This unequal arm size will increase data collected from the treatment arm, including adverse event rates. The trial is currently taking place at a single tertiary centre (Norfolk and Norwich University Hospital (NNUH), United Kingdom) and will remain open until July 2018. The intervention is methylphenidate, initially 10mg twice daily and increased to 20mg twice daily if appropriate, compared with an identical placebo. Participants and outcome assessors are blinded to the treatment received. Allocation is through an on-line system which allocates participants to methylphenidate or placebo and automatically informs the hospital pharmacy of the group allocated to allow dispensing of treatment whilst maintaining blinding. Participant flow through the trial is shown in figure 1.

Study participants are required to attend eight clinical visits over a 26-week period; one screening visit, a review at baseline, assessments for side-effects at two and four weeks, assessment of clinical outcomes at six, twelve, eighteen and twenty-four weeks. There is one post-trial assessment six weeks after discontinuing. Between visits, participants are contacted by phone-call to monitor for side-effects. Study visits and activities take place at the NNUH, although patients treated by other hospitals in the East of England region can be recruited if their centre is willing to act as a Participant Identification Centre (PIC site) and participants are willing to travel to the NNUH. The overview of study visits, including the assessments performed, is shown in table 1.

All participants must have a diagnosis of sarcoidosis. The full list of inclusion and exclusion criteria are shown in box 2. All eligible participants with sarcoidosis and fatigue (defined as a Fatigue Assessment Scale score more than 21) who are invited to participate in the study are provided with written information. Participants who agree to participate are required to give written informed consent. Initial contact with participants is during their attendance at the respiratory outpatient department or following referral from the patient's primary respiratory physician.

For peer review only

Interventions

A screening visit occurs two weeks prior to commencing study medications. This visit is used to collect written informed consent, baseline demographic data, measure blood pressure, pulse and perform a baseline electrocardiogram (ECG) and blood tests (renal function and electrolytes, liver function and full blood count). Female participants undertake a pregnancy test. Fatigue is quantified by FAS questionnaire; all participants must have a baseline FAS score of more than 21 points to participate. Obstructive sleep apnoea (OSA) is screened for using the STOP-Bang questionnaire. Participants scoring above 4 points undergo overnight pulse oximetry to determine if OSA is present, those with a desaturation index of greater than 15 being excluded and referred for consideration of treatment. Finally, potential participants wear an activity monitor (GENEActiv Original; Activinsights, Cambridgeshire, UK) on their non-dominant wrist for a seven-day period to measure daily activity levels prior to commencing therapy. Once baseline fatigue, co-morbidities, medications and blood tests have been checked to confirm no contraindication to participation, the subject is randomised.

When participants have been confirmed as eligible for participation in the FaST-MP study following their screening visit, they are invited back two weeks later to undertake the baseline visit which records pre-intervention questionnaire scores and spirometry. At this visit, participants complete fatigue scores (FAS score and the Functional Assessment of Chronic Illness Therapy – Fatigue (FACIT-F) questionnaire), quality of life scores (King's Sarcoidosis Questionnaire (KSQ), EuroQoL-5D-5L (EQ5D) and Short Form 36 (SF-36) questionnaires), depression symptom screening (Hospital Anxiety and Depression Score (HADS) questionnaire), sleep quality (Pittsburgh Sleep Quality Index (PSQI) questionnaire) and a custom costs questionnaire for health economic analysis. In addition, participants perform a modified shuttle walk test (MSWT) to determine exercise capacity and perform spirometry to measure baseline lung function. After these assessments have been

For peer review only

completed participants receive their first two-week supply of study medication, either methylphenidate 10mg twice daily or matched placebo.

The visit schedule over the first six weeks focuses on safety and adverse events, plus establishing a stable dose. After one week of therapy, participants receive a phone call from the study team to review potential adverse effects from the medication. A further week later the participant is seen for their first follow-up visit with safety measures taken (full blood count, renal function and liver function plus ECG) and review of side effects. If no adverse events have been identified then participants are uptitrated to the higher dose of 20mg methylphenidate twice daily if there has been insufficient clinical improvement in fatigue. Further safety visits (via phone on week 3 and 5, and with the study team for blood tests and ECG at week 4) subsequently occur; in the event of side effects emerging participants may be down-titrated to 10mg methylphenidate twice daily (See discontinuation criteria below for more details).

At six weeks participants return for a further safety visit, undergoing the same blood tests and a repeat ECG. In addition, the study questionnaires are repeated (FAS, FACIT-F, KSQ, EQ5D, SF36 and HADS), with the exception of the costs questionnaire which is performed at 12 and 24 weeks. Participants continue to receive phone calls to monitor for side effects every two weeks but are next seen face-to-face at 12 weeks, where all measures (safety bloods and ECG, adverse events, questionnaires including costs and PSQI, MSWT and spirometry) are repeated. In addition, participants wear a wrist-worn accelerometer for a further seven days to review daily activity levels and answers questions on sleep quality.

A further visit is undertaken at 18 weeks where participants undergo the same investigations as at 6 weeks, and finally again at 24 weeks where all study assessments are repeated, including an additional exit questionnaire collecting information about experiences of the trial and the medication. Participants on the higher dose of methylphenidate (20mg twice daily) are reduced to

For peer review only

10mg twice daily for two weeks; participants who complete the study on the lower dose discontinue all study medications at 24 weeks.

Following discontinuation of the study medication participants will undertake all questionnaires, excluding the cost questionnaire, and be assessed for side effects six weeks after discontinuing medications (30 weeks after commencing study medications). This will allow investigation of whether fatigue reported by participants returns to baseline level following discontinuing medication, or whether any benefit compared with baseline persists.

All participants are asked at the point of consenting to enter the study whether they would be willing to be approached about participating in focus groups following their completion of the study. Those who agreed to be approached about participating are offered the option to participate in focus groups following the completion of their trial participation; this is to investigate their experience and perception of their trial participation. There is a standardised topic guide (available from the authors) to direct discussions. All data is audio-recorded and then transcribed. It is not mandatory for all participants; we aim to have three groups of six to eight participants at completion of the study.

Outcomes

Primary and secondary outcomes are listed in box 1. Data collection points for each of the outcome measures are shown in table 1. The primary outcomes are related to feasibility, based upon recruitment (including number of patients who are eligible to participate), the number of participants that are retained at each point of the study, the number of participants providing data through to the end of the trial, and an estimate of treatment effect using change in the fatigue score measurements (FAS and FACIT-F) between methylphenidate and placebo groups which will influence future power calculations.

For peer review only

The output from this trial will influence the choice of primary outcome for future trials investigating sarcoidosis-associated fatigue. This is likely to be the FAS questionnaire as it is both symptom-specific for fatigue and has been validated in sarcoidosis cohorts previously(20). However, the interaction between changes in fatigue scores (both FAS and FACIT-F) and other measures, including depression and anxiety (HADS) and quality of life (KSQ, EQ5D and SF36), will be reviewed to determine how these measures change longitudinally with change in fatigue.

Additionally, a costs questionnaire will be administered to all participants. This is a custom-made instrument being piloted in this study which will look at costs incurred on a day-to-day basis by participants, including changes in out-of-pocket costs to participants relating to their health, health service usage and employment/working time. These methods can then be amended as required for appropriate cost estimation in a definitive trial in the future.

Concomitant therapies

Participants are able to receive any immunosuppressant drugs that would treat their sarcoidosis, but they are required to be on a stable dose of their medication for six weeks prior to study enrolment with no plan for a change in their dose during the trial. In the event that treatment is altered, either reducing or increasing the dose of existing therapy or starting additional medication for their sarcoidosis, this is recorded in their case report file.

There are a number of drugs which are contraindicated when receiving methylphenidate due to the risk of side-effects, and which participants are not permitted to receive during the trial. These include any tricyclic antidepressant (TCAs); any monoamine oxidase inhibitor (MAOI); all antipsychotics; sympathomimetics including phenylephrine, ephedrine or pseudoepherine; buprenorphine; tramadol; levodopa; clonidine; methylene blue; warfarin. Although both TCAs and

For peer review only

MAOIs are prohibited, selective serotonin inhibitors are allowed and have been used alongside methylphenidate safely previously(21).

Safety considerations

Methylphenidate is associated with side effects due to cardiac, neurological or psychiatric effects. The trial has been designed to minimise the risk of these complications occurring whilst receiving methylphenidate, both through the exclusion criteria and the visit schedule with safety monitoring. Cardiac side effects are screened for at baseline with ECG monitoring, and at every study visit thereafter. Any development of cardiac complications or ECG abnormalities, even if clinically asymptomatic, results in discontinuation of the study medication. Neurological and psychiatric problems are screened for, with any history of seizures (aside from febrile convulsions during childhood) or psychiatric disease (except depression) preventing patients from participating in the trial. Participants who are included in the trial are monitored at each visit for the emergence of any side effects, including seizures, abnormal behaviour or personality change.

A final safety consideration relates to methylphenidate being a controlled drug. The potential illicit use of methylphenidate risks participants selling or giving away medications to others. All participants will be informed about the illegal nature of these activities, and ask them to sign to indicate their understanding and agreement. Medication prescriptions are initially for two weeks, increased to six-week durations after six-weeks with participants bringing their remaining supply of medication to each visit for pill-count checking.

Discontinuation criteria

Immediate discontinuation will occur, regardless of the present dose, if participants develop a generalised rash or pruritis considered to be related to the intervention, develops an abnormality on

For peer review only

ECG, suffers chest pain consistent with angina, experiences severe anxiety or euphoria, personality change or bizarre behaviour, or if any psychiatric disease manifests during the trial. Other reasons for immediate discontinuation are seizures, severe hypertension (defined as a systolic blood pressure >180mmHg on two separate occasions or presenting with any features of malignant hypertension). Side effects which will lead to down-titration of the study drug in the first instance (if the participant is receiving the higher dose of 20mg methylphenidate twice daily) include nervousness or restlessness, nausea, indigestion, nasal stuffiness, cough, arthralgia, or anorexia. In consenting to the trial participants are agreeing to trial treatments, trial follow-up and data collection, including storage of anonymised data within a dataset available to other researchers from a data repository at the end of the trial. However, an individual can also choose to discontinue the study medication at any time.

Sample Size and Recruitment

The sample size of 30 patients was chosen in order to answer questions regarding recruitment, number of eligible participants and reasons for exclusion, and to pilot some elements of the trial, including the use of accelerometers measuring activity levels as part of the outcomes. This also ensures a minimum of 12 participants per group as per recommendations(22). This study is not powered to identify clinical efficacy, although an estimation of effect size between methylphenidate and placebo will enable power calculations to be performed for any future follow-on studies investigating methylphenidate.

All research activities occur at a single tertiary hospital site, although within this study we are using Participant Identification Centres (PICs) to improve recruitment by enabling a wider number of patients to be considered for inclusion within this study.

For peer review only

Data management and monitoring

A purpose-designed, secure password-protected electronic case report form is used for data entry throughout the trial, complying with data protection requirements on confidentiality and anonymity. The database enables screened patients to be randomised, linking with the local hospital pharmacy to securely randomise patients. The system is managed by the Norwich Clinical Trials Unit and can only be accessed by the chief investigator or appropriate members of the study team with delegated responsibility.

Throughout the trial monitoring will occur quarterly, carried out locally with oversight from the clinical trials unit and sponsor. A trial steering committee and safety committee monitor trial performance and adverse events. The two groups meet six-monthly and may terminate the trial if significant concerns become apparent, although formal stop criteria have not been set.

Data Analysis

Initial data analysis will evaluate pre-specified feasibility criteria. Firstly, a recruitment rate of 10% from our local sarcoidosis cohort is required to meet our recruitment aims; in the event of lower recruitment rates then consideration will need to be given to the ability to recruit the necessary number of sites, although this will be determined by the estimate of the mean difference in fatigue scores (effect size). The mean difference will be estimated using a general linear model for continuous outcomes, with an emphasis on estimation rather than hypothesis testing. Analysis will be based on an intention-to-treat approach. Adverse event data will be tabulated by category per group. No formal comparison will be made. Previous trails of methylphenidate have suggested an adverse events rate of less than 5% but these have not used the drug for more than 12 weeks (8 weeks in the previous trial involving patients with sarcoidosis-associated fatigue(17)). An adverse event rate of more than 10% during this 24-week trial would trigger consideration of the safety of

For peer review only

methylphenidate for longer periods and the suitability of using this agent. Finally, previous trials have managed a retention rate of more than 80% of participants; we will review the retention rate over this longer trial, aiming for over 60% retention to suggest a larger trial over such a period would be feasible. Understanding whether the other clinical measures being employed here (including the MSWT and accelerometer-measure of activity) are responsive to changes in fatigue scores and are therefore worth including in subsequent trials their success within this study will influence future trial design.

Power calculations for any subsequent trial will be performed using the data from change in the FAS score, which will inform sample size requirements, as well as data on drop-out rates through the trial.

Participant perception of undertaking the trial will be reviewed using the discussions in the focus groups. Participant views on the number of study visits, the intervention received (including the fact that it is a controlled drug), the specific measures used including the MSWT and activity monitor, and overall impression of participating in the trial (both positive and negative) will be reviewed following the focus groups, which will act as a debrief session for participants. This qualitative process evaluation has been recommended for use in the development of some RCTs and will enable optimisation of a future RCT through the experiences of participants(23).

Additionally, an exploratory analysis of the data will take place to investigate whether any change in fatigue and other measures is seen during the trial between groups, with appropriate adjustment for baseline values. The study is not powered for these clinical outcomes; this will be clearly stated in any outputs from these analyses.

Study Registration and Approvals

For peer review only

Approval from MHRA (EudraCT reference number 2016-000342-60) and REC (East of England – Cambridge Central, reference number 16/EE/0087) has been gained, with the trial adopted by the Health Research Authority (HRA). The study has been registered on a clinical trial database (clinicaltrials.gov), reference number NCT02643732. The study has been open for recruitment since October 2016 and will continue until July 2018.

Discussion

The FaST-MP study has been developed to inform decision-making regarding any future trial investigating the use of neurostimulants for the treatment of sarcoidosis-associated fatigue, a frequent and often challenging manifestation of chronic sarcoidosis. The results from this study will allow us to determine if progressing to a full-size trial using this drug is feasible, how many centres would be required, how the study would need to be structured and which outcome measures would be best employed to monitor progress throughout the trial. Piloting new outcome measures (costs questionnaire, activity monitors) during the course of this RCT will also enable us to evaluate these measures and their suitability for future studies.

Word Count: 3,350 words

For peer review only

Funding Statement:

This work is independent research supported by the National Institute of Health Research (NIHR Doctoral Research Fellowship, Dr Chris Atkins, DRF-2015-08-190). The views expressed in this publication are those of the authors and not necessarily those of the NHS, the National Institute for Health Research or the Department of Health.

Authors' Contributions:

CA, RF, AS, AC and AMW all made substantial contributions to the conception and design of the study. CA wrote the manuscript drafts. AMW made significant revisions to the manuscript. All authors read, amended and approved the final manuscript.

Competing interests statement:

None declared

For peer review only

Box 1 – Primary and Secondary Outcomes

Primary Outcome: Feasibility assessments

1. Recruitment rate and retention of participants
2. Reason for exclusion or withdrawal from the study
3. Number of participants suffering side-effects or requiring reduction of methylphenidate dose due to emergence of side effects, and the dose tolerated by greatest proportion of participants
4. Indication(s) of continuation of effect at stable dose during treatment period
5. Number of patients correctly using accelerometers
6. Acceptability of number of study visits and assessments
7. Acceptability of randomization
8. Acceptability of receiving a controlled drug
9. Mean change in fatigue score and standard deviation of change (for subsequent power calculations)
10. Patient perception of trial involvement (focus groups and exit questionnaire)

Secondary Outcomes: Exploratory assessment of clinical effects of methylphenidate over 24 weeks

1. Change in fatigue scores - Fatigue Assessment Scale (FAS) and Functional Assessment of Chronic Illness Therapy – Fatigue (FACIT-F)
2. Disease-specific quality of life – Kings Sarcoidosis Questionnaire (KSQ)
3. Depression and anxiety - Hospital Anxiety and Depression Scale (HADS)
4. Generic quality of life – EuroQoL-5D-5L (EQ5D) and Short Form 36 (SF-36)
5. Utilisation of health and social care - Cost questionnaire
6. Sleep quality - Pittsburgh Sleep Quality Index (PSQI); augmented with results from wrist-worn accelerometer data (awakenings)
7. Modified shuttle walk test distance - number of shuttles completed
8. Physical activity in free-living - mean accelerometer output (most active 5 hours, time in moderate or vigorous activity, time in sedentary behaviours)
9. Lung function - Spirometry (Forced Expiratory Volume in 1 second and Forced Vital Capacity)

For peer review only

Box 2 – Eligibility Criteria

Inclusion criteria:

1. A proven diagnosis of sarcoidosis – this is defined as either a biopsy-proven disease (non-caseating granulomas from a tissue biopsy), or a diagnosis of sarcoidosis agreed by an interstitial lung disease multidisciplinary team (ILD MDT) meeting.
2. Stable disease (treatment unchanged for 6 weeks, without anticipation of change in treatment during trial period)
3. Able to give informed consent
4. FAS score greater than 21 units

Exclusion criteria:

1. Evidence of co-existing obstructive sleep apnoea.
2. Documented history of significant cardiac disease (including cardiac sarcoid) OR associated disease which would increase risk of underlying coronary artery disease (cerebrovascular disease, previous stroke or peripheral vascular disease). Definitively treated cardiac disease e.g. previous myocardial infarction treated with stents or coronary artery bypass grafting with no ongoing symptoms is permitted.
3. Abnormal thyroid function (hyper or hypothyroidism)
4. History of seizures, excluding febrile convulsions whilst an infant.
5. Abnormal electrocardiogram (ECG) with evidence of arrhythmia (except first degree heart block which has been stable for 3 months).
6. Concomitant therapy with an excluded medication (see *concomitant therapies*)
7. Glaucoma or raised intra-ocular pressure for any reason.
8. Patients with established liver disease defined as Child-Pugh class B or C.
9. Documented medical history of psychiatric disorders (excluding depression)
10. History of drug-dependence or addiction at any time
11. Female participant who is pregnant, lactating or planning pregnancy during the course of the trial
12. Receiving an investigational drug or biological agent within 6 weeks (or 5 times the half-life if this is longer) prior to study entry.

For peer review only

Table 1 – Study Overview (SPIRIT template)

	STUDY PERIOD								
	Enrolment	Allocation	Post-allocation						Close-out
TIMEPOINT	*-2 weeks*	0 weeks	*2 weeks (+/- 3 days)*	*4 weeks (+/- 3 days)*	*6 weeks (+/- 3 days)*	*12 weeks (+/- 1 week)*	*18 weeks (+/- 1 week)*	*24 weeks (+/- 1 week)*	*+4-8 weeks*
**ENROLMENT:**									
Eligibility screen	X	X							
Informed consent	X								
Allocation		X							
**INTERVENTIONS:**									
Methylphenidate<br>*(X = uptitrate dose)*<br>*(O = drug dispensed)*		O	O<br>X	O	O	O	O		
Placebo<br>*(X = uptitrate dose)*<br>*(O = drug dispensed)*		O	O<br>X	O	O	O	O		
**ASSESSMENTS:**									
Safety bloods (FBC/LFT/U+Es)	X		X	X	X	X			
Safety questionnaire		X	X	X	X	X	X		
Pregnancy test	X	X							
ECG	X		X	X	X	X		X	
Spirometry		X				X		X	
Modified Shuttle Walk Test		X				X		X	
Accelerometer (7 days)	X					X		X	
**QUESTIONNAIRES:**									
- FAS	X	X	X	X	X	X	X	X	X
- FACIT-F		X	X	X	X	X	X	X	X
- HADS		X			X	X	X	X	X
- Short Form-36		X			X	X	X	X	X
- EQ5D		X			X	X	X	X	X
- KSQ		X			X	X	X	X	X
- Costs		X				X		X	
- PSQI		X				X		X	X
- Exit Questionnaire								X	
Focus group (post-trial)									X

In addition, all participants receive telephone calls at week 1, 3, 5, 8, 10, 14, 16, 10 and 22 to review safety (emergence of side effects)

For peer review only

Figure 1 – Patient flow through trial

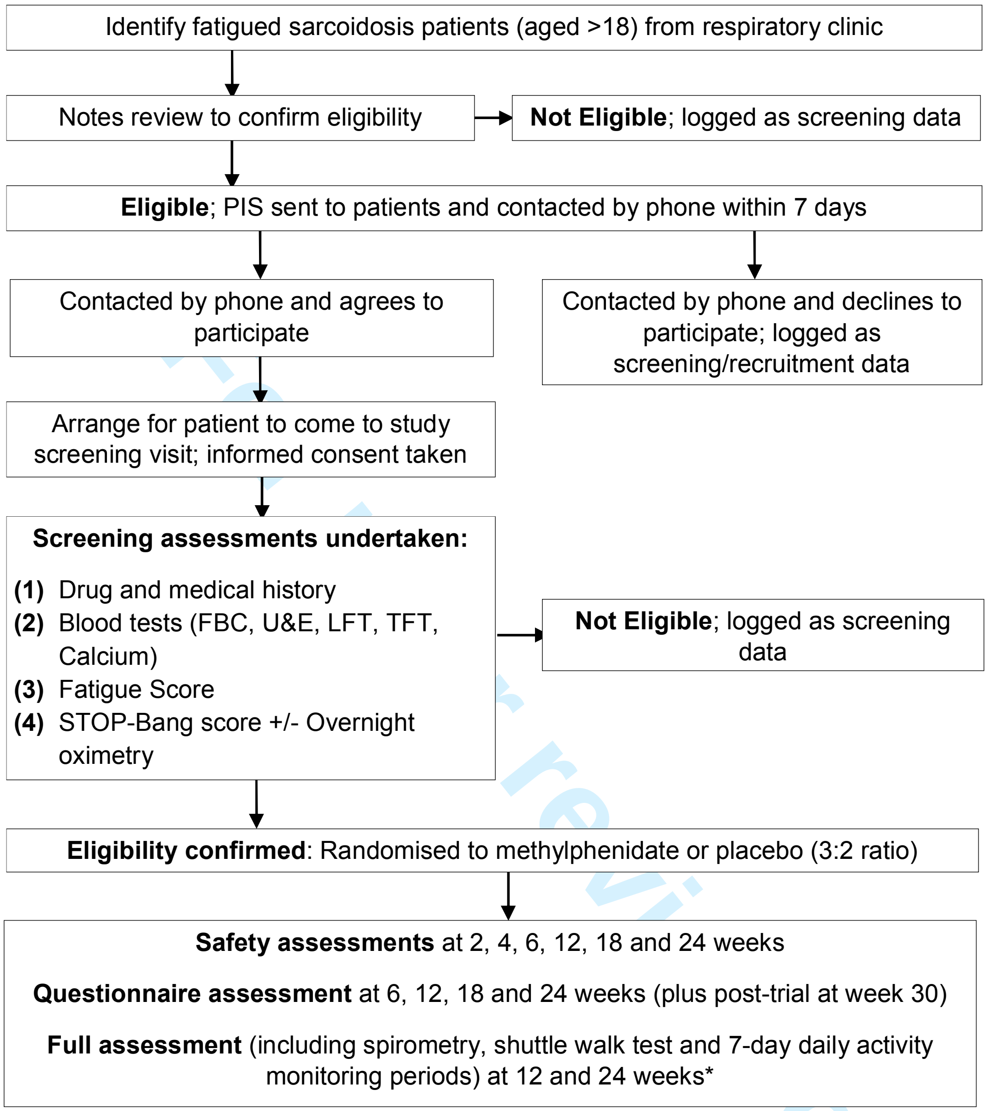

*Full descriptions of the assessments performed at each time point are shown in table 1.

For peer review only - http://bmjopen.bmj.com/site/about/guidelines.xhtml

References:

1. Gribbin J, Hubbard RB, Le Jeune I, Smith CJ, West J, Tata LJ. Incidence and mortality of idiopathic pulmonary fibrosis and sarcoidosis in the UK. Thorax. 2006;61(11):980-5.
2. Marcellis RGJ, Lenssen AF, Elfferich MDP, De Vries J, Kassim S, Foerster K, et al. Exercise capacity, muscle strength and fatigue in sarcoidosis. European Respiratory Journal. 2011;38(3):628-34.
3. Drent M, Lower EE, De Vries J. Sarcoidosis-associated fatigue. European Respiratory Journal. 2012;40(1):255-63.
4. Michielsen HJ, Drent M, Peros-Golubicic T, De Vries J. Fatigue is associated with quality of life in sarcoidosis patients. Chest. 2006;130(4):989-94.
5. Mostard RLM, Voo S, van Kroonenburgh MJPG, Verschakelen JA, Wijnen PAHM, Nelemans PJ, et al. Inflammatory activity assessment by F18 FDG-PET/CT in persistent symptomatic sarcoidosis. Respiratory medicine. 2011;105(12):1917-24.
6. Zheng L, Teschler H, Guzman J, Hubner K, Striz I, Costabel U. Alveolar macrophage TNF-alpha release and BAL cell phenotypes in sarcoidosis. American journal of respiratory and critical care medicine. 1995;152(3):1061-6.
7. Keller M, Mazuch J, Abraham U, Eom GD, Herzog ED, Volk HD, et al. A circadian clock in macrophages controls inflammatory immune responses. Proceedings of the National Academy of Sciences of the United States of America. 2009;106(50):21407-12.
8. Darko DF, Miller JC, Gallen C, White J, Koziol J, Brown SJ, et al. Sleep electroencephalogram delta-frequency amplitude, night plasma levels of tumor necrosis factor alpha, and human immunodeficiency virus infection. Proceedings of the National Academy of Sciences of the United States of America. 1995;92(26):12080-4.
9. De Kleijn WPE, Drent M, Vermunt JK, Shigemitsu H, De Vries J. Types of fatigue in sarcoidosis patients. Journal of Psychosomatic Research. 2011;71(6):416-22.
10. de Kleijn WPE, Drent M, De Vries J. Nature of fatigue moderates depressive symptoms and anxiety in sarcoidosis. British journal of health psychology. 2013;18(2):439-52.
11. Lal C, Medarov BI, Judson MA. Interrelationship Between Sleep-Disordered Breathing and Sarcoidosis. Chest. 2015;148(4):1105-14.
12. Fleischer M, Hinz A, Braehler E, Wirtz H, Bosse-Henck A. Factors Associated With Fatigue in Sarcoidosis. Respiratory Care. 2014;59(7):1086-94.
13. Atkins C, Wilson AM. Managing fatigue in sarcoidosis - A systematic review of the evidence. Chronic respiratory disease. 2016.
14. Minton O, Richardson A, Sharpe M, Hotopf M, Stone P. Drug therapy for the management of cancer-related fatigue. The Cochrane database of systematic reviews. 2010(7):CD006704.
15. Mendonca DA, Menezes K, Jog MS. Methylphenidate improves fatigue scores in Parkinson disease: a randomized controlled trial. Movement disorders : official journal of the Movement Disorder Society. 2007;22(14):2070-6.
16. Breitbart W, Rosenfeld B, Kaim M, Funesti-Esch J. A randomized, double-blind, placebo-controlled trial of psychostimulants for the treatment of fatigue in ambulatory patients with human immunodeficiency virus disease. Archives of internal medicine. 2001;161(3):411-20.
17. Lower EE, Harman S, Baughman RP. Double-blind, randomized trial of dexmethylphenidate hydrochloride for the treatment of sarcoidosis-associated fatigue. Chest. 2008;133(5):1189-95.
18. Lower EE, Malhotra A, Surdulescu V, Baughman RP. Armodafinil for Sarcoidosis-Associated Fatigue: A Double-Blind, Placebo-Controlled, Crossover Trial. Journal of pain and symptom management. 2013;45(2):159-69.
19. Baughman RP, Drent M, Culver DA, Grutters JC, Handa T, Humbert M, et al. ENDPOINTS FOR CLINICAL TRIALS OF SARCOIDOSIS. Sarcoidosis Vasculitis and Diffuse Lung Diseases. 2012;29(2):90-8.
20. De Vries J, Michielsen H, Van Heck GL, Drent M. Measuring fatigue in sarcoidosis: the Fatigue Assessment Scale (FAS). British journal of health psychology. 2004;9(Pt 3):279-91.

For peer review only

21. Lavretsky H, Reinlieb M, St Cyr N, Siddarth P, Ercoli LM, Senturk D. Citalopram, methylphenidate, or their combination in geriatric depression: a randomized, double-blind, placebo-controlled trial. The American journal of psychiatry. 2015;172(6):561-9.
22. Julious SA. Sample size of 12 per group rule of thumb for a pilot study. Pharmaceutical Statistics. 2005;4(4):287-91.
23. Murtagh MJ, Thomson RG, May CR, Rapley T, Heaven BR, Graham RH, et al. Qualitative methods in a randomised controlled trial: the role of an integrated qualitative process evaluation in providing evidence to discontinue the intervention in one arm of a trial of a decision support tool. Quality & safety in health care. 2007;16(3):224-9.

For peer review only

BMJ Open

Feasibility study of a randomised controlled trial to investigate the treatment of sarcoidosis-associated fatigue with methylphenidate (FaST-MP): a study protocol

Journal:	*BMJ Open*
Manuscript ID	bmjopen-2017-018532.R1
Article Type:	Protocol
Date Submitted by the Author:	25-Oct-2017
Complete List of Authors:	Atkins, Christopher; University of East Anglia Norwich Medical School, Department of Medicine; University of East Anglia Norwich Medical School, Department of Medicine<br>Fordham, Richard; University of East Anglia Norwich Medical School, Department of Medicine<br>Clark, Allan; University of East Anglia Norwich Medical School, Department of Medicine<br>Stockl, Andrea; University of East Anglia Norwich Medical School, Department of Medicine<br>Jones, Andrew; University of East Anglia, Norwich Medical School<br>Wilson, Andrew; University of East Anglia Norwich Medical School, Department of Medicine
**Primary Subject Heading**:	Respiratory medicine
Secondary Subject Heading:	Research methods, Pharmacology and therapeutics
Keywords:	Interstitial lung disease < THORACIC MEDICINE, Sarcoidosis, Fatigue

SCHOLARONE™
Manuscripts

Feasibility study of a randomised controlled trial to investigate the treatment of sarcoidosis-associated fatigue with methylphenidate (FaST-MP): a study protocol

[1,2]C Atkins[a], [1]R Fordham, [1]A Clarke, [1]A Stockl, [2]A P Jones, [1,2]A M Wilson

[1]Norwich Medical School, University of East Anglia, Norwich, Norfolk, UK, NR4 7TJ

[2]Norfolk and Norwich University Hospital, Norwich, Norfolk, UK, NR4 7UY

[a]Corresponding author: Dr Chris Atkins, Norwich Medical School, University of East Anglia, Norwich, Norfolk, UK, NR4 7TJ

E-mail contact: c.atkins@uea.ac.uk

For peer review only

For peer review only - http://bmjopen.bmj.com/site/about/guidelines.xhtml

Abstract

Introduction

Fatigue is a frequent and troublesome manifestation of chronic sarcoidosis. This symptom can be debilitating and difficult to treat, with poor response to treatment. Symptomatic management with neurostimulants such as methylphenidate is a possible treatment option. The use of such treatment strategies is not without precedent and has been trialled in cancer-related fatigue. Their use in sarcoidosis requires further evaluation before it can be recommended for clinical practice.

Methods and Analysis

The Fatigue and Sarcoidosis – Treatment with Methylphenidate (FaST-MP) study is a randomised, controlled, parallel-arm, feasibility trial of methylphenidate for the treatment of sarcoidosis-associated fatigue. Patients are eligible if they have a diagnosis of sarcoidosis, significant fatigue (measured using the Fatigue Assessment Scale) and have stable disease. Up to 30 participants will be randomly assigned to either methylphenidate (20mg twice daily) or identical placebo in a 3:2 ratio for 24 weeks. The primary objective is to collect data determining the feasibility of a future study powered to determine the clinical efficacy of methylphenidate for sarcoidosis-associated fatigue. The trial is presently open and will continue until July 2018.

Ethics and Dissemination

Ethical approval for the study was granted by the Cambridge Central Research Ethics Committee on 21 June 2016 (reference 16/EE/0087) and was approved and sponsored by the Norfolk and Norwich University Hospital (reference 190280). Clinical Trial Authorisation (EudraCT number 2016-000342-60) from the MHRA was granted on 19 April 2016. Results will be presented at relevant conferences and submitted to appropriate journals following trial closure and analysis.

Trial registration: NCT02643732

For peer review only - http://bmjopen.bmj.com/site/about/guidelines.xhtml

Article Summary

Strengths and limitations of this study:

- Twenty-four week duration of receiving methylphenidate or placebo
- Variety of outcome measures included to inform future study design
- Exercise measurements during study done both in clinic (modified shuttle walk test) and using objective measures of exercise during free-living (wrist-worn activity monitors)
- Small participant numbers will not prove clinical efficacy of methylphenidate but does allow appropriate future study design to be determined
- No stratification for age or sex due to small number of participants

For peer review only - http://bmjopen.bmj.com/site/about/guidelines.xhtml
For peer review only

Introduction

Sarcoidosis is a granulomatous, multi-system disease with an incidence of 5 per 100,000 person-years, typically affecting patients of working age(1). Fatigue has been described as a "core symptom" of sarcoidosis and is present in up to 80% of patients (2, 3). The presence of fatigue has shown to adversely affect quality of life(4). Sarcoidosis-associated fatigue has multiple possible aetiologies. These include subclinical disease activity(5), increased circulating tumour necrosis factor (TNF)-alpha levels(6), disturbed circadian rhythm(7) and sleep(8), small-fibre neuropathy(9), depression(10), sleep-disordered breathing problems(11), treatment-related side-effects (12), or a combination of any of these factors.

Role of neurostimulants in treating fatigue

Current evidence for treatments of sarcoidosis-associated fatigue are weak, with further work required(13). The use of methylphenidate for fatigue is not without precedent, having been trialled in cancer-related fatigue and recommended as effective for symptomatic relief after meta-analysis by the Cochrane collaboration(14). There is also evidence for effect in fatigue associated with Parkinson's disease(15) and HIV(16). In all cases, the treatment has only been trialled for a short period of time (up to 12 weeks). Neurostimulants have been trialled for symptomatic relief of fatigue associated with sarcoidosis. Both methylphenidate(17) and modafinil(18) have been trialled in patients with significant fatigue, though in only a very small sample (10 patients trialled methylphenidate and 15 trialled modafinil) over only eight weeks, a period of time that does not reflect the typical period of time that these agents may be used for in clinical practice. Despite initial evidence for the use of methylphenidate in sarcoidosis-associated fatigue being published almost ten years ago no further studies have been published; the reasons for this are unclear when sarcoidosis-associated fatigue remains a significant clinical issue without established treatment strategies. Questions remain about ongoing treatment effect, side effects during protracted

For peer review only

For peer review only - http://bmjopen.bmj.com/site/about/guidelines.xhtml

treatment, and whether patients would continue to take the medication over a longer period. For these reasons this feasibility study has been undertaken prior expanding to a large, multi-centre RCT.

Rationale for this pilot trial

High quality evidence is required to establish the clinical efficacy and long-term tolerability of methylphenidate for the treatment of sarcoidosis-associated fatigue. Change in fatigue scores measured by a validated questionnaire measure of fatigue across a longer treatment period of six months would be able to address the issue of efficacy, but the evidence to support a large-scale randomised controlled trial (RCT), or determine the optimal design of such an RCT, is presently insufficient.

Furthermore, although recommendations from the World Association of Sarcoidosis and Other Granulomatous diseases (WASOG) recommend measuring fatigue in trials involving sarcoidosis patients using a validated questionnaire(19), we plan to also investigate the effect on activity levels with treatment using wrist-worn accelerometers alongside typical questionnaire measures. Outputs from these activity monitors appear to correlate with fatigue scores, both in terms of reduced activity and increased time in sedentary behaviours (unpublished data), and may give further insight into how improvements in fatigue affect day-to-day living in patients. To plan a sufficiently large RCT we propose a pilot placebo-controlled RCT to inform the design of a future, definitive trial investigating management of fatigue.

Objectives

The primary hypothesis is to determine the feasibility and design of a future, large-scale RCT investigating the use of methylphenidate to treat sarcoidosis-associated fatigue and provide sustained benefit over a six-month period. In addition, it will pilot the use of wrist-worn, tri-axial

For peer review only - http://bmjopen.bmj.com/site/about/guidelines.xhtml

accelerometer-based activity monitors to monitor change in activity and sedentary behaviours during treatment alongside validated questionnaire-based fatigue scores.

Methods

Participants

This study is a randomised, double-blind, placebo-controlled single-centre parallel-group trial with randomisation of up to 30 patients in a ratio of 3:2 in favour of methylphenidate. This unequal arm size will increase data collected from the treatment arm, including adverse event rates. The trial is currently taking place at a single tertiary centre (Norfolk and Norwich University Hospital (NNUH), United Kingdom) and will remain open until July 2018. Participants are identified through the respiratory clinic at the NNUH. The intervention is methylphenidate, initially 10mg twice daily and increased to 20mg twice daily if appropriate, compared with an identical placebo. Participants and outcome assessors are blinded to the treatment received. Allocation is through an on-line system which allocates participants to methylphenidate or placebo and automatically informs the hospital pharmacy of the group allocated to allow dispensing of treatment whilst maintaining blinding. Participant flow through the trial is shown in figure 1.

Study participants are required to attend eight clinical visits over a 26-week period; one screening visit, a review at baseline, assessments for side-effects at two and four weeks, assessment of clinical outcomes at six, twelve, eighteen and twenty-four weeks. There is one post-trial assessment six weeks after discontinuing. Between visits participants are contacted by phone-call to monitor for side-effects. Study visits and activities take place at the NNUH, although patients treated by other hospitals in the East of England region can be recruited if their centre is willing to act as a Participant Identification Centre (PIC site) and participants are willing to travel to the NNUH. The overview of study visits, including the assessments performed, is shown in table 1.

For peer review only - http://bmjopen.bmj.com/site/about/guidelines.xhtml
For peer review only

All participants must have a diagnosis of sarcoidosis. The full list of inclusion and exclusion criteria are shown in box 1. All eligible participants with sarcoidosis and fatigue (defined as a Fatigue Assessment Scale score more than 21) who are invited to participate in the study are provided with written information. Participants who agree to participate are required to give written informed consent. Initial contact with participants is during their attendance at the respiratory outpatient department or following referral from the patient's primary respiratory physician.

Interventions

A screening visit occurs two weeks prior to commencing study medications. This visit is used to collect written informed consent, baseline demographic data, list current medications (including current and prior treatment for sarcoidosis), measure blood pressure, pulse and perform a baseline electrocardiogram (ECG) and blood tests (renal function and electrolytes, liver function and full blood count). Female participants undertake a pregnancy test. Clinical data is reviewed for manifestations of sarcoidosis, to determine whether extra-pulmonary disease is present. Fatigue is quantified by FAS questionnaire; all participants must have a baseline FAS score of more than 21 points to participate. Obstructive sleep apnoea (OSA) is screened for using the STOP-Bang questionnaire. Participants scoring above 4 points undergo overnight pulse oximetry to determine if OSA is present, those with a desaturation index of greater than 15 being excluded and referred for consideration of treatment. Finally, potential participants wear an activity monitor (GENEActiv Original; Activinsights, Cambridgeshire, UK) on their non-dominant wrist for a seven-day period to measure daily activity levels prior to commencing therapy. Once baseline fatigue, co-morbidities, medications and blood tests have been checked to confirm no contraindication to participation, the subject is randomised.

When participants have been confirmed as eligible for participation in the FaST-MP study following their screening visit, they are invited back two weeks later to undertake the baseline visit which

For peer review only - http://bmjopen.bmj.com/site/about/guidelines.xhtml
For peer review only

records pre-intervention questionnaire scores and spirometry. At this visit, participants complete fatigue scores (FAS score and the Functional Assessment of Chronic Illness Therapy – Fatigue (FACIT-F) questionnaire), quality of life scores (King's Sarcoidosis Questionnaire (KSQ), EuroQoL-5D-5L (EQ5D) and Short Form 36 (SF-36) questionnaires), depression symptom screening (Hospital Anxiety and Depression Score (HADS) questionnaire), sleep quality (Pittsburgh Sleep Quality Index (PSQI) questionnaire) and a custom costs questionnaire for health economic analysis. In addition, participants perform a modified shuttle walk test (MSWT) to determine exercise capacity and perform spirometry to measure baseline lung function. After these assessments have been completed participants receive their first two-week supply of study medication, either methylphenidate 10mg twice daily or matched placebo.

The visit schedule over the first six weeks focuses on safety and adverse events, plus establishing a stable dose. After one week of therapy, participants receive a phone call from the study team to review potential adverse effects from the medication. A further week later the participant is seen for their first follow-up visit with safety measures taken (full blood count, renal function and liver function plus ECG) and review of side effects. If no adverse events have been identified then participants are titrated up to the higher dose of 20mg methylphenidate twice daily if there has been insufficient clinical improvement in fatigue. Further safety visits (via phone on week 3 and 5, and with the study team for blood tests and ECG at week 4) subsequently occur; in the event of side effects emerging participants may be down-titrated to 10mg methylphenidate twice daily (see discontinuation criteria below for more details).

At six weeks participants return for a further safety visit, undergoing the same blood tests and a repeat ECG. In addition, the study questionnaires are repeated (FAS, FACIT-F, KSQ, EQ5D, SF36 and HADS), with the exception of the costs questionnaire which is performed at 12 and 24 weeks. Participants continue to receive phone calls to monitor for side effects every two weeks but are next seen face-to-face at 12 weeks, where all measures (safety bloods and ECG, adverse events,

For peer review only - http://bmjopen.bmj.com/site/about/guidelines.xhtml
For peer review only

questionnaires including costs and PSQI, MSWT and spirometry) are repeated. In addition, participants wear a wrist-worn accelerometer for a further seven days to review daily activity levels and answers questions on sleep quality.

A further visit is undertaken at 18 weeks where participants undergo the same investigations as at 6 weeks, and finally again at 24 weeks where all study assessments are repeated, including an additional exit questionnaire collecting information about experiences of the trial and the medication. Participants on the higher dose of methylphenidate (20mg twice daily) are reduced to 10mg twice daily for two weeks; participants who complete the study on the lower dose discontinue all study medications at 24 weeks.

Following discontinuation of the study medication participants will undertake all questionnaires, excluding the cost questionnaire, and be assessed for side effects six weeks after discontinuing medications (30 weeks after commencing study medications). This will allow investigation of whether fatigue returns to baseline level following discontinuation of medication, or whether any benefit compared with baseline persists.

All participants are asked at the point of consenting to enter the study whether they would be willing to be approached about participating in focus groups following their completion of the study. Those who agreed to be approached about participating are offered the option to participate in focus groups following the completion of their trial participation; this is to investigate their experience and perception of their trial participation. There is a standardised topic guide to direct discussions (available from the authors). All data is audio-recorded and then transcribed. It is not mandatory for all participants; we aim to have three groups of six to eight participants at completion of the study.

Outcomes

For peer review only - http://bmjopen.bmj.com/site/about/guidelines.xhtml
For peer review only

Primary and secondary outcomes are listed in box 2. Data collection points for each of the outcome measures are shown in table 1. The primary outcomes are related to feasibility, based upon recruitment (including number of patients who are eligible to participate), the number of participants that are retained at each point of the study, the number of participants providing data through to the end of the trial, and an estimate of treatment effect using change in the fatigue score measurements (FAS and FACIT-F) between methylphenidate and placebo groups which will influence future power calculations.

The output from this trial will influence the choice of primary outcome for future trials investigating sarcoidosis-associated fatigue. This is likely to be the FAS questionnaire as it is both symptom-specific for fatigue and has been validated in sarcoidosis cohorts previously(20). However, the interaction between changes in fatigue scores (both FAS and FACIT-F) and other measures, including depression and anxiety (HADS) and quality of life (KSQ, EQ5D and SF36), will be reviewed to determine how these measures change longitudinally with change in fatigue.

Additionally, a costs questionnaire will be administered to all participants. This is a custom-made instrument being piloted in this study which will look at costs incurred on a day-to-day basis by participants, including changes in out-of-pocket costs to participants relating to their health, health service usage and employment/working time. These methods can then be amended as required for appropriate cost estimation in a definitive trial in the future.

Concomitant therapies

Participants are able to receive any immunosuppressant drugs that would treat their sarcoidosis, but they are required to be on a stable dose of their medication for six weeks prior to study enrolment with no planned change in dose during the trial. In the event that treatment is altered, either

For peer review only - http://bmjopen.bmj.com/site/about/guidelines.xhtml

reducing or increasing the dose of existing therapy or starting additional medication for their sarcoidosis, this is recorded in their case report file.

There are a number of drugs which are contraindicated when receiving methylphenidate due to the risk of side-effects; participants are not permitted to receive these during the trial. These include any tricyclic antidepressant (TCAs); any monoamine oxidase inhibitor (MAOI); all antipsychotics; sympathomimetics including phenylephrine, ephedrine or pseudoepherine; buprenorphine; tramadol; levodopa; clonidine; methylene blue; warfarin. Although both TCAs and MAOIs are prohibited, selective serotonin inhibitors are allowed and have been used alongside methylphenidate safely previously(21).

Safety considerations

Methylphenidate is associated with side effects due to cardiac, neurological or psychiatric effects. The trial has been designed to minimise the risk of these complications occurring whilst receiving methylphenidate, both through the exclusion criteria and the visit schedule with safety monitoring. Cardiac side effects are screened for at baseline with ECG monitoring, and at every study visit thereafter. Any development of cardiac complications or ECG abnormalities, even if clinically asymptomatic, results in discontinuation of the study medication. Neurological and psychiatric problems are screened for, with any history of seizures (aside from febrile convulsions during childhood) or psychiatric disease (except depression) preventing patients from participating in the trial. Participants who are included in the trial are monitored at each visit for the emergence of any side effects, including seizures, abnormal behaviour or personality change.

A final safety consideration relates to methylphenidate being a controlled drug. The potential illicit use of methylphenidate risks participants selling or giving away medications to others. All participants will be informed about the illegal nature of these activities, and asked to sign a

For peer review only - http://bmjopen.bmj.com/site/about/guidelines.xhtml
For peer review only

document to indicate their understanding and agreement. Medication prescriptions are initially for two weeks, increased to six-weeks after the third visit (at six weeks) with participants bringing their remaining supply of medication to each visit for pill-count checking.

Discontinuation criteria

Immediate discontinuation will occur, regardless of the present dose, if participants develop a generalised rash or pruritis considered to be related to the intervention, develops an abnormality on ECG, suffers chest pain consistent with angina, experiences severe anxiety or euphoria, personality change or bizarre behaviour, or if any psychiatric disease manifests during the trial. Other reasons for immediate discontinuation are seizures, severe hypertension (defined as a systolic blood pressure >180mmHg on two separate occasions or presenting with any features of malignant hypertension). Side effects which will lead to down-titration of the study drug in the first instance (if the participant is receiving the higher dose of 20mg methylphenidate twice daily) include nervousness or restlessness, significant nausea or indigestion, nasal stuffiness, cough, arthralgia, or anorexia. Participants can also choose to discontinue the study medication at any time for any reason. In consenting to the trial participants are agreeing to trial treatments, trial follow-up and data collection, including storage of anonymised data within a dataset available to other researchers from a data repository at the end of the trial.

For peer review only

Sample Size and Recruitment

The sample size of 30 patients was chosen in order to answer questions regarding recruitment, number of eligible participants and reasons for exclusion, and to pilot some elements of the trial, including the use of accelerometers measuring activity levels as part of the outcomes. This also ensures a minimum of 12 participants per group as per recommendations(22). This study is not

For peer review only - http://bmjopen.bmj.com/site/about/guidelines.xhtml

powered to identify clinical efficacy, although an estimation of effect size between methylphenidate and placebo will enable power calculations to be performed for any future follow-on studies investigating methylphenidate.

All research activities occur at a single tertiary hospital site, although within this study we are using Participant Identification Centres (PICs) to improve recruitment by enabling a wider number of patients to be considered for inclusion within this study.

Data management and monitoring

A purpose-designed, secure password-protected electronic case report form is used for data entry throughout the trial, complying with data protection requirements on confidentiality and anonymity. The database enables screened patients to be randomised, linking with the local hospital pharmacy to securely randomise patients via e-mail. This maintains blinding of the clinical team, research team and investigators, and patients. Allocation is determined based upon block randomisation in blocks of five with stratification by severity of fatigue (FAS 22-34 and FAS 35-50). The system is managed by the Norwich Clinical Trials Unit and can only be accessed by the chief investigator or appropriate members of the study team with delegated responsibility.

Throughout the trial monitoring will occur quarterly, carried out locally with oversight from the clinical trials unit and sponsor. A trial steering committee and safety committee monitor trial performance and adverse events. The two groups meet six-monthly and may terminate the trial if significant concerns become apparent, although formal stop criteria have not been set.

Data Analysis

Initial data analysis will evaluate pre-specified feasibility criteria. Firstly, a recruitment rate of 10% from our local sarcoidosis cohort is required to meet our recruitment aims; in the event of lower

For peer review only - http://bmjopen.bmj.com/site/about/guidelines.xhtml
For peer review only

recruitment rates then consideration will need to be given to the ability to recruit the necessary number of sites, although this will be determined by the estimate of the mean difference in fatigue scores (effect size). The mean difference will be estimated using a general linear model for continuous outcomes, with an emphasis on estimation rather than hypothesis testing. Analysis will be based on an intention-to-treat approach. Adverse event data will be tabulated by category per group. No formal comparison will be made. Previous trails of methylphenidate have suggested an adverse events rate of less than 5% but these have not used the drug for more than 12 weeks (8 weeks in the previous trial involving patients with sarcoidosis-associated fatigue(17)). An adverse event rate of more than 10% during this 24-week trial would trigger consideration of the safety of methylphenidate for longer periods and the suitability of using this agent. Finally, previous trials have managed a retention rate of more than 80% of participants; we will review the retention rate over this longer trial, aiming for over 60% retention to suggest a larger trial over such a period would be feasible. Understanding whether the other clinical measures being employed here (including the MSWT and accelerometer-measure of activity) are responsive to changes in fatigue scores, and therefore worth including in subsequent trials, will determine if they should be included in future studies.

Power calculations for any subsequent trial will be performed using the data from change in the FAS score, which will inform sample size requirements, as well as data on drop-out rates through the trial.

Participant perception of undertaking the trial will be reviewed using the discussions in the focus groups. Participant views on the number of study visits, the intervention received (including the fact that it is a controlled drug), the specific measures used including the MSWT and activity monitor, and overall impression of participating in the trial (both positive and negative) will be reviewed following the focus groups, which will act as a debrief session for participants. This qualitative

For peer review only - http://bmjopen.bmj.com/site/about/guidelines.xhtml

process evaluation has been recommended for use in the development of some RCTs and will enable optimisation of a future RCT through the experiences of participants(23).

Exploratory analysis of the data will take place to investigate whether any change in fatigue or other measures is seen during the trial between groups, with appropriate adjustment for baseline values. The study is not powered for these clinical outcomes; this will be clearly stated in any outputs from these analyses.

Study Registration and Approvals

Approval from MHRA (EudraCT reference number 2016-000342-60) and REC (East of England – Cambridge Central, reference number 16/EE/0087) has been gained, with the trial adopted by the Health Research Authority (HRA). The study has been registered on a clinical trial database (clinicaltrials.gov), reference number NCT02643732. The study has been open for recruitment since October 2016 and will continue until July 2018.

Discussion

The FaST-MP study has been developed to inform decision-making regarding any future trial investigating the use of neurostimulants for the treatment of sarcoidosis-associated fatigue, a frequent and often challenging manifestation of chronic sarcoidosis. The results from this study will allow us to determine if progressing to a full-size trial using this drug is feasible, how many centres would be required, how the study would need to be structured and which outcome measures would be best employed to monitor progress throughout the trial. Piloting new outcome measures (costs questionnaire, activity monitors) during the course of this RCT will also enable us to evaluate these measures and their suitability for future studies.

For peer review only - http://bmjopen.bmj.com/site/about/guidelines.xhtml
For peer review only

Word Count: 3,491 words

For peer review only

For peer review only - http://bmjopen.bmj.com/site/about/guidelines.xhtml

Funding Statement:

This work is independent research supported by the National Institute of Health Research (NIHR Doctoral Research Fellowship, Dr Chris Atkins, DRF-2015-08-190). The views expressed in this publication are those of the authors and not necessarily those of the NHS, the National Institute for Health Research or the Department of Health.

Authors' Contributions:

CA, RF, AS, AC, APJ and AMW all made substantial contributions to the conception and design of the study. CA wrote the manuscript drafts. AMW made significant revisions to the manuscript. All authors read, amended and approved the final manuscript.

Competing interests statement:

None declared

For peer review only

For peer review only - http://bmjopen.bmj.com/site/about/guidelines.xhtml

Box 1 – Eligibility Criteria

Inclusion criteria:

1. A proven diagnosis of sarcoidosis – this is defined as either a biopsy-proven disease (non-caseating granulomas from a tissue biopsy), or a diagnosis of sarcoidosis agreed by an interstitial lung disease multidisciplinary team (ILD MDT) meeting.
2. Stable disease (treatment unchanged for 6 weeks, without anticipation of change in treatment during trial period)
3. Able to give informed consent
4. FAS score greater than 21 units

Exclusion criteria:

1. Evidence of co-existing obstructive sleep apnoea.
2. Documented history of significant cardiac disease (including cardiac sarcoid) OR associated disease which would increase risk of underlying coronary artery disease (cerebrovascular disease, previous stroke or peripheral vascular disease). Definitively treated cardiac disease e.g. previous myocardial infarction treated with stents or coronary artery bypass grafting with no ongoing symptoms is permitted.
3. Abnormal thyroid function (hyper or hypothyroidism)
4. History of seizures, excluding febrile convulsions whilst an infant.
5. Abnormal electrocardiogram (ECG) with evidence of arrhythmia (except first degree heart block which has been stable for 3 months).
6. Concomitant therapy with an excluded medication (see *concomitant therapies*)
7. Glaucoma or raised intra-ocular pressure for any reason.
8. Patients with established liver disease defined as Child-Pugh class B or C.
9. Documented medical history of psychiatric disorders (excluding depression)
10. History of drug-dependence or addiction at any time
11. Female participant who is pregnant, lactating or planning pregnancy during the course of the trial
12. Receiving an investigational drug or biological agent within 6 weeks (or 5 times the half-life if this is longer) prior to study entry.

For peer review only

For peer review only - http://bmjopen.bmj.com/site/about/guidelines.xhtml

Box 2 – Primary and Secondary Outcomes

Primary Outcome: Feasibility assessments

1. Recruitment rate and retention of participants
2. Reason for exclusion or withdrawal from the study
3. Number of participants suffering side-effects or requiring reduction of methylphenidate dose due to emergence of side effects, and the dose tolerated by greatest proportion of participants
4. Indication(s) of continuation of effect at stable dose during treatment period
5. Number of patients correctly using accelerometers
6. Acceptability of number of study visits and assessments
7. Acceptability of randomization
8. Acceptability of receiving a controlled drug
9. Mean change in fatigue score and standard deviation of change (for subsequent power calculations)
10. Patient perception of trial involvement (focus groups and exit questionnaire)

Secondary Outcomes: Exploratory assessment of clinical effects of methylphenidate over 24 weeks

1. Change in fatigue scores - Fatigue Assessment Scale (FAS) and Functional Assessment of Chronic Illness Therapy – Fatigue (FACIT-F)
2. Disease-specific quality of life – Kings Sarcoidosis Questionnaire (KSQ)
3. Depression and anxiety - Hospital Anxiety and Depression Scale (HADS)
4. Generic quality of life – EuroQoL-5D-5L (EQ5D) and Short Form 36 (SF-36)
5. Utilisation of health and social care - Cost questionnaire
6. Sleep quality - Pittsburgh Sleep Quality Index (PSQI); augmented with results from wrist-worn accelerometer data (awakenings)
7. Modified shuttle walk test distance - number of shuttles completed
8. Physical activity in free-living - mean accelerometer output (most active 5 hours, time in moderate or vigorous activity, time in sedentary behaviours)
9. Lung function - Spirometry (Forced Expiratory Volume in 1 second and Forced Vital Capacity)

For peer review only

For peer review only - http://bmjopen.bmj.com/site/about/guidelines.xhtml

Table 1 – Study Overview (SPIRIT template)

	STUDY PERIOD								
	Enrolment	Allocation	Post-allocation						Close-out
TIMEPOINT	*-2 weeks*	0 weeks	*2 weeks (+/- 3 days)*	*4 weeks (+/- 3 days)*	*6 weeks (+/- 3 days)*	*12 weeks (+/- 1 week)*	*18 weeks (+/- 1 week)*	*24 weeks (+/- 1 week)*	*+4-8 weeks*
**ENROLMENT:**									
Eligibility screen	X	X							
Informed consent	X								
Allocation		X							
**INTERVENTIONS:**									
Methylphenidate (X = uptitrate dose) (O = drug dispensed) [line from 0 weeks to 24 weeks]		O	O X	O	O	O	O		
Placebo (X = uptitrate dose) (O = drug dispensed) [line from 0 weeks to 24 weeks]		O	O X	O	O	O	O		
**ASSESSMENTS:**									
Safety bloods (FBC/LFT/U+Es)	X		X	X	X	X			
Safety questionnaire		X	X	X	X	X	X		
Pregnancy test	X	X							
ECG	X		X	X	X	X		X	
Spirometry		X				X		X	
Modified Shuttle Walk Test		X				X		X	
Accelerometer (7 days)	X					X		X	
**QUESTIONNAIRES:**									
- FAS	X	X	X	X	X	X	X	X	X
- FACIT-F		X	X	X	X	X	X	X	X
- HADS		X			X	X	X	X	X
- Short Form-36		X			X	X	X	X	X
- EQ5D		X			X	X	X	X	X
- KSQ		X			X	X	X	X	X
- Costs		X				X		X	
- PSQI		X				X		X	X
- Exit Questionnaire								X	
Focus group (post-trial)									X

In addition, all participants receive telephone calls at week 1, 3, 5, 8, 10, 14, 16, 10 and 22 to review safety (emergence of side effects)

For peer review only

For peer review only - http://bmjopen.bmj.com/site/about/guidelines.xhtml

References:

1. Gribbin J, Hubbard RB, Le Jeune I, Smith CJ, West J, Tata LJ. Incidence and mortality of idiopathic pulmonary fibrosis and sarcoidosis in the UK. Thorax. 2006;61(11):980-5.
2. Marcellis RGJ, Lenssen AF, Elfferich MDP, De Vries J, Kassim S, Foerster K, et al. Exercise capacity, muscle strength and fatigue in sarcoidosis. European Respiratory Journal. 2011;38(3):628-34.
3. Drent M, Lower EE, De Vries J. Sarcoidosis-associated fatigue. European Respiratory Journal. 2012;40(1):255-63.
4. Michielsen HJ, Drent M, Peros-Golubicic T, De Vries J. Fatigue is associated with quality of life in sarcoidosis patients. Chest. 2006;130(4):989-94.
5. Mostard RLM, Voo S, van Kroonenburgh MJPG, Verschakelen JA, Wijnen PAHM, Nelemans PJ, et al. Inflammatory activity assessment by F18 FDG-PET/CT in persistent symptomatic sarcoidosis. Respiratory medicine. 2011;105(12):1917-24.
6. Zheng L, Teschler H, Guzman J, Hubner K, Striz I, Costabel U. Alveolar macrophage TNF-alpha release and BAL cell phenotypes in sarcoidosis. American journal of respiratory and critical care medicine. 1995;152(3):1061-6.
7. Keller M, Mazuch J, Abraham U, Eom GD, Herzog ED, Volk HD, et al. A circadian clock in macrophages controls inflammatory immune responses. Proceedings of the National Academy of Sciences of the United States of America. 2009;106(50):21407-12.
8. Darko DF, Miller JC, Gallen C, White J, Koziol J, Brown SJ, et al. Sleep electroencephalogram delta-frequency amplitude, night plasma levels of tumor necrosis factor alpha, and human immunodeficiency virus infection. Proceedings of the National Academy of Sciences of the United States of America. 1995;92(26):12080-4.
9. De Kleijn WPE, Drent M, Vermunt JK, Shigemitsu H, De Vries J. Types of fatigue in sarcoidosis patients. Journal of Psychosomatic Research. 2011;71(6):416-22.
10. de Kleijn WPE, Drent M, De Vries J. Nature of fatigue moderates depressive symptoms and anxiety in sarcoidosis. British journal of health psychology. 2013;18(2):439-52.
11. Lal C, Medarov BI, Judson MA. Interrelationship Between Sleep-Disordered Breathing and Sarcoidosis. Chest. 2015;148(4):1105-14.
12. Fleischer M, Hinz A, Braehler E, Wirtz H, Bosse-Henck A. Factors Associated With Fatigue in Sarcoidosis. Respiratory Care. 2014;59(7):1086-94.
13. Atkins C, Wilson AM. Managing fatigue in sarcoidosis - A systematic review of the evidence. Chronic respiratory disease. 2016.
14. Minton O, Richardson A, Sharpe M, Hotopf M, Stone P. Drug therapy for the management of cancer-related fatigue. The Cochrane database of systematic reviews. 2010(7):CD006704.
15. Mendonca DA, Menezes K, Jog MS. Methylphenidate improves fatigue scores in Parkinson disease: a randomized controlled trial. Movement disorders : official journal of the Movement Disorder Society. 2007;22(14):2070-6.
16. Breitbart W, Rosenfeld B, Kaim M, Funesti-Esch J. A randomized, double-blind, placebo-controlled trial of psychostimulants for the treatment of fatigue in ambulatory patients with human immunodeficiency virus disease. Archives of internal medicine. 2001;161(3):411-20.
17. Lower EE, Harman S, Baughman RP. Double-blind, randomized trial of dexmethylphenidate hydrochloride for the treatment of sarcoidosis-associated fatigue. Chest. 2008;133(5):1189-95.
18. Lower EE, Malhotra A, Surdulescu V, Baughman RP. Armodafinil for Sarcoidosis-Associated Fatigue: A Double-Blind, Placebo-Controlled, Crossover Trial. Journal of pain and symptom management. 2013;45(2):159-69.
19. Baughman RP, Drent M, Culver DA, Grutters JC, Handa T, Humbert M, et al. ENDPOINTS FOR CLINICAL TRIALS OF SARCOIDOSIS. Sarcoidosis Vasculitis and Diffuse Lung Diseases. 2012;29(2):90-8.
20. De Vries J, Michielsen H, Van Heck GL, Drent M. Measuring fatigue in sarcoidosis: the Fatigue Assessment Scale (FAS). British journal of health psychology. 2004;9(Pt 3):279-91.

For peer review only - http://bmjopen.bmj.com/site/about/guidelines.xhtml

21. Lavretsky H, Reinlieb M, St Cyr N, Siddarth P, Ercoli LM, Senturk D. Citalopram, methylphenidate, or their combination in geriatric depression: a randomized, double-blind, placebo-controlled trial. The American journal of psychiatry. 2015;172(6):561-9.
22. Julious SA. Sample size of 12 per group rule of thumb for a pilot study. Pharmaceutical Statistics. 2005;4(4):287-91.
23. Murtagh MJ, Thomson RG, May CR, Rapley T, Heaven BR, Graham RH, et al. Qualitative methods in a randomised controlled trial: the role of an integrated qualitative process evaluation in providing evidence to discontinue the intervention in one arm of a trial of a decision support tool. Quality & safety in health care. 2007;16(3):224-9.

For peer review only

For peer review only - http://bmjopen.bmj.com/site/about/guidelines.xhtml

Figure Legends:

Box 1 – Eligibility criteria

Box 2 – Primary and secondary objectives

Figure 1 – Patient flow through trial

For peer review only

For peer review only - http://bmjopen.bmj.com/site/about/guidelines.xhtml

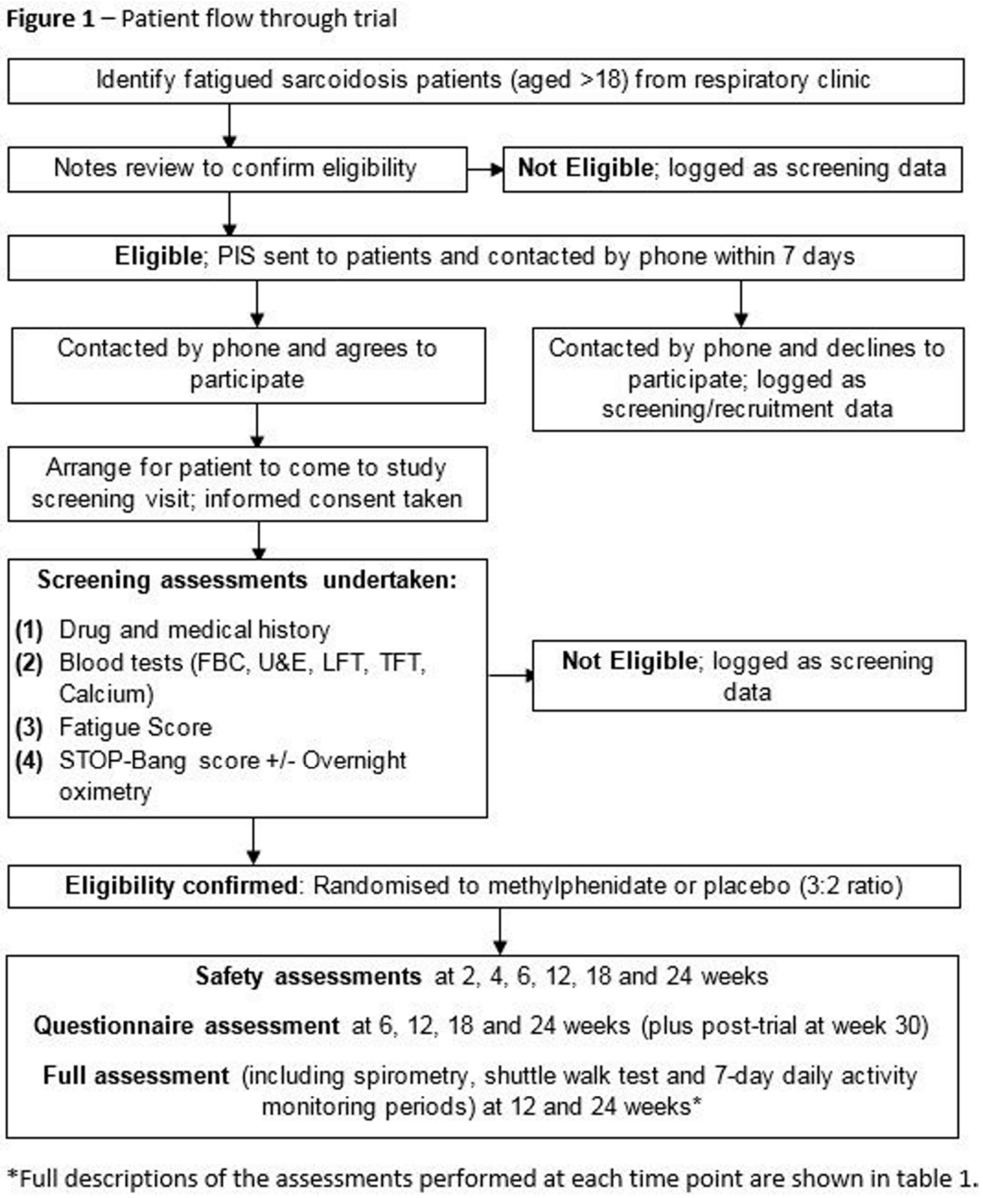

Figure 1 – Patient flow through trial

90x110mm (300 x 300 DPI)

CONSORT 2010 checklist of information to include when reporting a pilot or feasibility trial*

Section/Topic	Item No	Checklist item	Reported on page No
**Title and abstract**			
	1a	Identification as a pilot or feasibility randomised trial in the title	1
	1b	Structured summary of pilot trial design, methods, results, and conclusions (for specific guidance see CONSORT abstract extension for pilot trials)	2
**Introduction**			
Background and objectives	2a	Scientific background and explanation of rationale for future definitive trial, and reasons for randomised pilot trial	4,5
	2b	Specific objectives or research questions for pilot trial	5,6
**Methods**			
Trial design	3a	Description of pilot trial design (such as parallel, factorial) including allocation ratio	6
	3b	Important changes to methods after pilot trial commencement (such as eligibility criteria), with reasons	n/a
Participants	4a	Eligibility criteria for participants	10,11
	4b	Settings and locations where the data were collected	6
	4c	How participants were identified and consented	6
Interventions	5	The interventions for each group with sufficient details to allow replication, including how and when they were actually administered	7,8,9
Outcomes	6a	Completely defined prespecified assessments or measurements to address each pilot trial objective specified in 2b, including how and when they were assessed	8,9, box 1
	6b	Any changes to pilot trial assessments or measurements after the pilot trial commenced, with reasons	n/a
	6c	If applicable, prespecified criteria used to judge whether, or how, to proceed with future definitive trial	Not set, discussed on pages 13,14
Sample size	7a	Rationale for numbers in the pilot trial	12
	7b	When applicable, explanation of any interim analyses and stopping guidelines	n/a
Randomisation:			
Sequence generation	8a	Method used to generate the random allocation sequence	13
	8b	Type of randomisation(s); details of any restriction (such as blocking and block size)	13
Allocation concealment	9	Mechanism used to implement the random allocation sequence (such as sequentially numbered containers), describing any steps taken to conceal the sequence until interventions were assigned	13

For peer review only

mechanism			
Implementation	10	Who generated the random allocation sequence, who enrolled participants, and who assigned participants to interventions	13
Blinding	11a	If done, who was blinded after assignment to interventions (for example, participants, care providers, those assessing outcomes) and how	6,13
	11b	If relevant, description of the similarity of interventions	6
Statistical methods	12	Methods used to address each pilot trial objective whether qualitative or quantitative	13,14
**Results**			
Participant flow (a diagram is strongly recommended)	13a	For each group, the numbers of participants who were approached and/or assessed for eligibility, randomly assigned, received intended treatment, and were assessed for each objective	20 (Fig 1)
	13b	For each group, losses and exclusions after randomisation, together with reasons	n/a
Recruitment	14a	Dates defining the periods of recruitment and follow-up	6
	14b	Why the pilot trial ended or was stopped	n/a (ongoing)
Baseline data	15	A table showing baseline demographic and clinical characteristics for each group	n/a (ongoing)
Numbers analysed	16	For each objective, number of participants (denominator) included in each analysis. If relevant, these numbers should be by randomised group	n/a (ongoing)
Outcomes and estimation	17	For each objective, results including expressions of uncertainty (such as 95% confidence interval) for any estimates. If relevant, these results should be by randomised group	n/a (ongoing)
Ancillary analyses	18	Results of any other analyses performed that could be used to inform the future definitive trial	13
Harms	19	All important harms or unintended effects in each group (for specific guidance see CONSORT for harms)	n/a
	19a	If relevant, other important unintended consequences	n/a
**Discussion**			
Limitations	20	Pilot trial limitations, addressing sources of potential bias and remaining uncertainty about feasibility	n/a (ongoing)
Generalisability	21	Generalisability (applicability) of pilot trial methods and findings to future definitive trial and other studies	n/a (ongoing)
Interpretation	22	Interpretation consistent with pilot trial objectives and findings, balancing potential benefits and harms, and considering other relevant evidence	n/a (ongoing)
	22a	Implications for progression from pilot to future definitive trial, including any proposed amendments	n/a (ongoing)
**Other information**			
Registration	23	Registration number for pilot trial and name of trial registry	15
Protocol	24	Where the pilot trial protocol can be accessed, if available	n/a – for publication
Funding	25	Sources of funding and other support (such as supply of drugs), role of funders	16
	26	Ethical approval or approval by research review committee, confirmed with reference number	1

For peer review only

Citation: Eldridge SM, Chan CL, Campbell MJ, Bond CM, Hopewell S, Thabane L, et al. CONSORT 2010 statement: extension to randomised pilot and feasibility trials. BMJ. 2016;355.
*We strongly recommend reading this statement in conjunction with the CONSORT 2010, extension to randomised pilot and feasibility trials, Explanation and Elaboration for important clarifications on all the items. If relevant, we also recommend reading CONSORT extensions for cluster randomised trials, non-inferiority and equivalence trials, non-pharmacological treatments, herbal interventions, and pragmatic trials. Additional extensions are forthcoming: for those and for up to date references relevant to this checklist, see www.consort-statement.org.

For peer review only
